# Influenza A(H3N2) Variant Virus Outbreak at Three Fairs — Maryland, 2017

**DOI:** 10.15585/mmwr.mm6742a1

**Published:** 2018-10-26

**Authors:** Monique M. Duwell, David Blythe, Michael W. Radebaugh, Erin M. Kough, Brian Bachaus, David A. Crum, Keith A. Perkins, Lenee Blanton, C. Todd Davis, Yunho Jang, Amy Vincent, Jennifer Chang, Dianna E. Abney, Lisa Gudmundson, Meenakshi G. Brewster, Larry Polsky, David C. Rose, Katherine A. Feldman

**Affiliations:** ^1^Epidemic Intelligence Service, CDC; ^2^Maryland Department of Health; ^3^Maryland Department of Agriculture; ^4^Influenza Division, National Center for Immunization and Respiratory Diseases, CDC; ^5^Virus and Prion Research Unit, National Animal Disease Center, Agricultural Research Service, U.S. Department of Agriculture; ^6^Charles County Department of Health, White Plains, Maryland; ^7^Frederick County Health Department, Frederick, Maryland; ^8^St. Mary’s County Health Department, Leonardtown, Maryland; ^9^Calvert County Health Department, Prince Frederick, Maryland;^10^Anne Arundel County Department of Health, Annapolis, Maryland.

On September 17, 2017, the Maryland Department of Agriculture (MDA) was notified by fair and 4-H officials of ill swine at agricultural fair A, held September 14–17. That day, investigation of the 107 swine at fair A revealed five swine with fever and signs of upper respiratory tract illness. All five respiratory specimens collected from these swine tested positive for influenza A virus at the MDA Animal Health Laboratory, and influenza A(H3N2) virus was confirmed in all specimens by the U.S. Department of Agriculture National Veterinary Services Laboratory (NVSL). On September 18, MDA was notified by fair and 4-H officials that swine exhibitors were also ill. MDA alerted the Maryland Department of Health (MDH). A joint investigation with MDH and the local health department was started and later broadened to Maryland agricultural fairs B (September 13–17) and C (September 15–23). In total, 76 persons underwent testing for variant influenza, and influenza A(H3N2) variant (A(H3N2)v) virus infection was identified in 40 patients with exposure to swine at these fairs (Figure), including 30 (75%) who had more than one characteristic putting them at high risk for serious influenza complications; 24 (60%) of these were children aged <5 years. Twenty-six (65%) patients reported direct contact with swine (i.e., touching swine or swine enclosure), but 14 (35%) reported only indirect contact (e.g., walking through a swine barn). Two children required hospitalization; all patients recovered. This outbreak highlights the risk, particularly among children, for contracting variant influenza virus at agricultural fairs after direct or indirect swine contact. Publicizing CDC’s recommendation that persons at high risk for serious influenza complications avoid pigs and swine barns might help prevent future variant influenza outbreaks among vulnerable groups (*1*).

**FIGURE:**
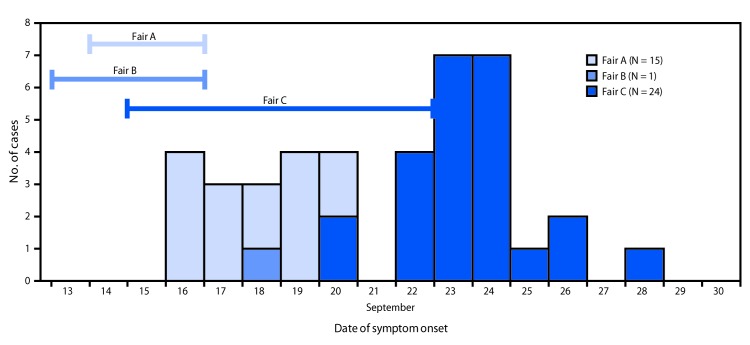
Human influenza A(H3N2) variant virus infections (N = 40), by date of symptom onset and associated agricultural fair — Maryland, September 2017

## Investigation and Results

Public health and agricultural officials met with fair and 4-H club leaders and swine exhibitors and identified an initial group of eight ill persons associated with fair A. They telephoned these persons and coordinated collection of nasopharyngeal (NP) swab specimens. On September 20, seven of eight NP specimens tested presumptively positive for A(H3N2)v virus by real-time reverse transcription–polymerase chain reaction (RT-PCR) testing at MDH Laboratories Administration.

A suspected case of variant influenza was defined as influenza-like illness (ILI) (fever and cough or sore throat) among persons with swine exposure ≤7 days before symptom onset. Case-finding activities included contacting swine exhibitors through in-person meetings, telephone calls, and e-mail. Through media outreach, persons with recent swine exposure and ILI were advised to seek medical testing. MDH sent an e-mail alert to clinicians and published an Epi-X* alert call for cases. NP swabs from ill persons were collected at local health departments, clinics, and hospitals; initial testing was performed at the MDH Laboratories Administration.

On September 21, MDH was notified by the local health department of an ill person with swine exposure at fair B, and specimen collection was conducted in the patient’s home state of Delaware, in coordination with the Delaware Division of Public Health. No ill swine were detected at fair B, and no swine influenza testing was performed at this fair. The ill person had no other swine exposure and no sick contacts. On September 23, ill swine and ill persons with swine exposure at fair C were reported by fair officials. Among 294 swine at fair C, 11 were found to have fever and signs of upper respiratory tract illness. All 11 respiratory specimens collected from swine at fair C tested positive for influenza A at MDA Animal Health Laboratory and influenza A(H3N2) virus was confirmed in all specimens by NVSL. On September 27, MDH reported the first human A(H3N2)v virus presumptively positive cases associated with fairs B and C.

In total, 80 fair attendees reporting ILI were identified; 76 underwent influenza testing. Forty (52.6%) persons tested presumptively positive for influenza A(H3N2)v virus infection, including 39 Maryland residents who had NP swabs tested at the Maryland Laboratories Administration and one Delaware resident whose NP swab was tested at the Delaware Public Health Laboratory. All were confirmed to contain influenza A(H3N2)v virus by real-time RT-PCR testing and genetic sequencing analysis performed at CDC. Telephone interviews were conducted with all patients who tested presumptively positive using a novel influenza A virus case report form to collect demographic, health, and exposure information.

All patients reported attending one of three Maryland fairs (A [15], B [one], and C [24]); 52.5% were male. Patient age ranged from 9 months to 79 years; 37 (92.5%) were aged <15 years. Overall, 30 (75%) patients were at high risk for complications from influenza, including 24 aged <5 years, one aged ≥65 years, and six with a chronic medical condition. Twenty-six (65%) patients reported direct contact with swine. Fourteen (35%) patients reported only indirect contact with swine.

The median incubation period from last swine exposure to symptom onset was 2.5 days (range = 1–6 days). The most commonly reported signs and symptoms were fever (92.5%), cough (92.5%), and sore throat (40%). Eight patients reported vaccination against seasonal influenza in the past year. Two children were hospitalized; one of whom had an underlying medical condition and reported direct contact with swine. The other, admitted to an intensive care unit, was previously healthy and was wheeled in a stroller through a swine barn, but had no direct swine contact. Both recovered; there were no deaths.

## Laboratory Data

Viruses were isolated from two swine each at fairs A and C from clinical samples submitted from the MDA Animal Health Laboratory to NVSL. Whole-genome sequencing was performed on the four swine viruses at NVSL in accordance with guidelines stated in the National Surveillance Plan for influenza in pigs (*2*). All eight gene segments of the viruses were amplified using standard methods. Sequences were submitted to GenBank (*3*).^†^

Whole-genome sequences from human cases were generated for 34 viruses representing strains from each fair. Hemagglutinin (HA) and neuraminidase (NA) sequences only were available for one additional A(H3N2)v virus.^§^ For phylogenetic analyses, nucleotide sequences were downloaded from GenBank for swine and from the Global Initiative on Sharing All Influenza Data (GISAID) for human A(H3N2)v viruses. Swine and human viruses from the Maryland fairs included in the investigation were highly similar to one another in each gene segment (>99% identity) and formed monophyletic clades in each of the gene trees. The Maryland A(H3N2)v and swine influenza viruses were also similar to other swine exhibit–associated variant cases detected during 2017 from other states (*4*).

## Public Health Response

In accordance with Maryland law and MDA’s standard operating procedures for swine influenza, steps were taken to minimize transmission of influenza virus among swine and from swine to humans. At fair A, MDA allowed market swine to go to slaughter, and remaining nonmarket swine (i.e., breeder pigs) were quarantined on the fairgrounds until 7 days after the last swine showed signs of influenza illness. At fair C, MDA sent some breeder pigs home, increased surveillance of remaining pigs for illness, and closed swine exhibits to the public; when swine illness was later detected, market swine were allowed to go to slaughter and remaining swine were quarantined on the fairgrounds. The Maryland Secretary of Agriculture canceled swine exhibits at the final two fairs of the 2017 Maryland fair season.

Public health officials issued press releases, conducted media interviews, and created a variant influenza virus website to educate the public about risk, prevention, and treatment. A letter was sent advising preventive measures to schools and child care providers, particularly for field trips where children might be exposed to swine. In counties with canceled swine exhibits, MDA, MDH, and 4-H leaders held meetings with persons who had planned to exhibit swine to arrange for the safe sale and processing of market swine.

## Discussion

This outbreak highlights the ongoing public health risk for variant influenza at agricultural fairs. In past outbreaks, most variant influenza virus infections and related hospitalizations have been among children (*4*,*5*). The proportion of ill children in this outbreak, including both hospitalized patients, underscores the vulnerability of children to variant influenza virus and its potential life-threatening complications.

Seventy-five percent of patients in this outbreak, including both hospitalized patients, were at high risk for influenza complications. CDC recommends that persons at high risk for serious influenza complications avoid pigs and swine barns (*1*). Increasing public awareness about these recommendations (e.g., through signage posted at swine barn entrances) might decrease variant influenza virus infection among these groups. Although immunization with seasonal influenza vaccine does not provide protection against infection with variant viruses, the low number of patients who reported seasonal influenza vaccination underscores the need to reinforce that seasonal influenza vaccination is recommended for all persons aged ≥6 months (*6*).

This outbreak also highlights the need to expand monitoring for swine and human respiratory illness to concurrent or upcoming agricultural fairs within the same region if swine influenza virus is detected at one fair. Influenza A viruses are endemic and circulate among most swine populations in North America (*7*). It is common for swine to be exhibited at multiple fairs (such as county and state fairs) and even in multiple states. Strategies to prevent potential influenza transmission at other ongoing or upcoming regional agricultural fairs might include enhanced surveillance for swine illness and public educational campaigns about variant influenza. In situations where the risk of variant influenza is high, such as when a variant influenza case at a nearby fair has been detected, closing ongoing swine exhibits to the public and canceling upcoming swine exhibits might also be considered.

Finally, this outbreak highlights the need for a One Health approach to investigating and responding to variant influenza virus outbreaks, including the application of both swine and human infection control measures, as well as collaboration between agricultural, environmental, and public health agencies on surveillance and communications strategies. This approach is collaborative, multisectoral, and transdisciplinary, used at the local, regional, national, and global levels, and recognizes the interconnection between humans, animals, plants, and their shared environment.

**Summary**

**What is already known about this topic?**

Outbreaks of variant influenza have occurred in agricultural fair settings in the United States.

**What is added by this report?**

In September 2017, 40 cases of influenza A(H3N2) variant virus infection were identified among persons with swine exposure at one of three Maryland agricultural fairs. Thirty cases (75%) occurred among persons at high risk for serious influenza complications. Thirty-five percent of patients reported only indirect swine contact.

**What are the implications for public health practice?**

Increased public education that groups at high risk for influenza complications should avoid pigs and swine barns is needed. When swine influenza virus is detected at one fair, enhanced surveillance should extend to all fairs in the region.

## Footnotes

* https://www.cdc.gov/mmwr/epix/epix.html.

† Nucleotide accession numbers: MG193837–MG193844 for A/swine/Maryland/A01764002/2017(H3N2); MG193875–MG193882 for A/swine/Maryland/A01764005/2017(H3N2); MG193883–MG193890 for A/swine/Maryland/A01764022/2017(H3N2); and MG193799–MG193806 for A/swine/Maryland/A01764024/2017(H3N2) for swine viruses.

§ GISAID accession numbers: EPI_ISL_294259 to EPI_ISL_294289, EPI_ISL_294291, EPI_ISL_294294, EPI_ISL_294296, EPI_ISL_304787.
